# The Jack and Jill Adaptive Working Memory Task: Construction, Calibration and Validation

**DOI:** 10.1371/journal.pone.0262200

**Published:** 2022-01-27

**Authors:** Elina Tsigeman, Sebastian Silas, Klaus Frieler, Maxim Likhanov, Rebecca Gelding, Yulia Kovas, Daniel Müllensiefen

**Affiliations:** 1 Sirius University of Science and Technology, Sochi, Russia; 2 Department of Psychology, Tomsk State University, Tomsk, Russia; 3 Department of Psychology, Goldsmiths University of London, London, United Kingdom; 4 Hochschule für Musik, Theater und Medien, Hannover, Germany; 5 Max-Planck-Institute of Empirical Aesthetics, Frankfurt a.M., Germany; 6 ITMO University, Saint Petersburg, Russia; 7 Macquarie University, Sydney, Australia; Liverpool John Moores University, UNITED KINGDOM

## Abstract

Visuospatial working memory (VSWM) is essential to human cognitive abilities and is associated with important life outcomes such as academic performance. Recently, a number of reliable measures of VSWM have been developed to help understand psychological processes and for practical use in education. We sought to extend this work using Item Response Theory (IRT) and Computerised Adaptive Testing (CAT) frameworks to construct, calibrate and validate a new adaptive, computerised, and open-source VSWM test. We aimed to overcome the limitations of previous instruments and provide researchers with a valid and freely available VSWM measurement tool. The Jack and Jill (JaJ) VSWM task was constructed using explanatory item response modelling of data from a sample of the general adult population (Study 1, *N* = 244) in the UK and US. Subsequently, a static version of the task was tested for validity and reliability using a sample of adults from the UK and Australia (Study 2, *N* = 148) and a sample of Russian adolescents (Study 3, *N* = 263). Finally, the adaptive version of the JaJ task was implemented on the basis of the underlying IRT model and evaluated with another sample of Russian adolescents (Study 4, *N* = 239). JaJ showed sufficient internal consistency and concurrent validity as indicated by significant and substantial correlations with established measures of working memory, spatial ability, non-verbal intelligence, and academic achievement. The findings suggest that JaJ is an efficient and reliable measure of VSWM from adolescent to adult age.

## Introduction

The term working memory (WM) first appeared in Miller, Galanter, and Pribham [1] where the authors described it as a storage system “where plans can be retained temporarily when they are being formed, or transformed, or executed”. More recently, it has been described as the “brain’s conductor” [2], because of its vital role in human learning. WM has also been described as a “cognitive primitive” [3], which constrains the acquisition and deployment of most intellectual abilities [4].

Several theoretical WM frameworks have been presented in the literature, including *the embedded-processes model* [5]; *the biologically based model* [6]; *the attention control model* [7]; and *the time-based resource-sharing model* [8]. To date, one of the most influential WM models is *the multi-component model* [9–11] which describes WM as a limited capacity system that enables the temporary storage and manipulation of information. According to this model, WM contains four components: the *phonological loop (*a slave subsystem that stores verbal information), the *visuospatial sketchpad (visuospatial working memory*, VSWM; a slave subsystem that stores and manipulates visuospatial information), the *episodic buffer* (a mechanism for multimodal information exchange between WM and other types of memory, e.g., long-term memory) and the *central executive* (which enables attentional focus, manipulation, and decision-making).

WM correlates substantially with measures of intelligence (e.g., [12] and also predicts a range of salient life outcomes such as reading comprehension [13, 14], domain expertise [15] and academic achievement [16, 17]. In particular, VSWM capacity appears to be a predictor across a wide spectrum of achievements in different domains, showing moderate correlations with reading performance [18], achievement in English [19], maths, and related areas such as geometry and informatics [14, 20] as well as science [21] in schoolchildren.

### Structure and measurement of visuospatial working memory

VSWM is believed to consist of several components, which can be differentiated by distinct experimental tasks. Logie [22] described VSWM as a tripartite model consisting of a passive *visual cache* for maintaining information, an active *inner scribe* for enabling rehearsal, and a *central executive* system, which in turn integrates and controls the other two facets. Each of the three components showed distinctive developmental patterns in Logie and Pearson [23].

Della Sala, Gray, Baddeley, Allamano, and Wilson [24] suggested another two different distinct components of VSWM. Their model distinguishes between visual and spatial components, with the spatial component also potentially consisting of a spatial-sequential subset. The visual component enables maintaining the appearance of objects (shape, colour, etc. as assessed by e.g., the odd-one-out task) and location (e.g., Corsi block-tapping test; [25]). The spatial component enables identifying changes between stimuli (e.g., *The Visual Patterns Test*; [26]). A similar model differentiating between visual, spatial (simultaneous and sequential) and visuospatial complex span components was suggested by the data in Mammarella and colleagues [27]. Other research suggests somewhat different structures when considering WM more broadly. For example, one study performed a confirmatory factor analysis on 10 visuospatial and 3 verbal WM tasks, with the best model containing four passive components for storage (verbal, visual, simultaneous-spatial, and sequential-spatial) and one active component for processing (central executive) [28]. Active processes are suggested to involve the manipulation of information in addition to information maintenance, while passive processes include only maintenance of information that is not modified after encoding.

Many individual differences tasks which have been employed to measure aspects of VSWM have some notable limitations. In particular, they lack a close relationship to a cognitive model of WM that would bolster the task’s construct validity (e.g., [29]). They may also share the problem of task impurity–measuring several WM components at once. Some research (e.g., [30]) addressed this issue by assessing performance on multiple WM tasks and then adopting a latent variable approach to extract the common variance among the tasks. Yet tasks may still involve other cognitive abilities, such as long-term memory encoding or retrieval [31]. Additionally, while there are some well-established visuospatial tests (e.g., in the Woodcock-Johnson III (WJ III) Tests of Cognitive Abilities); [32], sometimes they might be too expensive to be used by researchers and educators.

Furthermore, many traditional measures of VSWM do not require concurrent processing and storage of visuospatial information, which is the simultaneous engagement of the central executive and visuospatial sketchpad, according to the Baddeley and Hitch WM model. To address this gap in the test literature, Shah and Miyake [33] proposed a new spatial span task using a dual-task paradigm, as typical for experimental WM research. The task requires participants to mentally rotate sequentially presented visual shapes (i.e., letters), while also encoding the shapes’ spatial positions in the correct order. The number of correctly recalled location sequences represents the participant’s VSWM score. The task showed high reliability (*Cronbach’s* α = .80) and loaded strongly onto a spatial WM factor in their original study [33]. These results were replicated in a later study [34], where the test was named ‘letter rotation task’ and loaded on a complex storage-plus-processing VSWM factor (standardized factor loading = .64), as opposed to a simple-storage visuospatial short-term memory factor or an executive function factor. In sum, the letter rotation task represents an easy-to-understand dual-task paradigm that requires processing and storage of two different attributes (orientation and location) of the same visual shape. It has good psychometric properties, is easy to score, and task difficulty can be easily manipulated via the length of sequences presented.

However, the letter rotation task requires the recognition of letter shapes and hence is not suitable for use with young children, illiterate individuals, or participants with severe reading impairments. Therefore, Alloway and colleagues created a new version of the task (the ‘Mister X’ task) for use with individuals from early childhood (5 years) to adulthood (69 years), as part of *The Automated Working Memory Assessment* (AWMA; [35, 36]). The Mister X task is considered visually more appealing (to children) due to its use of two illustrated human figures that are presented simultaneously on the screen, each holding a ball in one hand. One Mister X figure is rotated, and participants are required to judge whether both Mister X figures hold the ball in the same hand. In addition, participants must remember the spatial location of the ball of the right figure and recall all ball locations in the correct order, after the end of the sequence of trials. The Mister X task showed good psychometric benchmarks (test-retest reliability, *r* = .77, [36]) and a single clear loading on a VSWM factor for children as well as adults in an independent validation and application study [37]. The evidence of the task’s reliability and validity for use with adults and children is robust and made the Mister X task a good candidate for further development into a computerised adaptive task with automated item generation.

### The present study

The objective of this study is to further adapt the Mister X task to improve the practicality, quality, and efficiency of VSWM measurement. We emphasise that the Mister X task and its predecessors have already been validated and were considered VSWM tasks in the literature. Consequently, our objective is to further validate the task in different settings. For instance, high WM abilities are often associated with obtaining high levels of expertise in given domains, like music [38–40]. Yet, the Mister X task we reproduced was only validated in populations of young children from the general population. Hence, we wanted to extend the domain further to see whether JaJ could also be validated within the context of high performing/specialist populations (Studies 3 and 4).

Here, we document the development and validation of a new adaptive, computerised and open-source WM task–Jack and Jill—based on modern psychometric techniques.

We expanded on the Mister X task in a number of ways. Firstly, in an attempt to engage younger participants, the style of the graphics was chosen to be more cartoon-like. Further, instead of one single character in the task, we created two characters of different genders (i.e., Jack and Jill). This may make the task more widely relatable, especially to younger participants who may find a Mister X character more abstract than the nursery rhyme characters Jack and Jill.

The second change concerns the design and calibration of the test based on item response theory (IRT, see e.g., [41] for an introduction). IRT provides a principled and flexible measurement framework for the Jack and Jill task. The IRT framework allows the computation of estimates of item difficulties and person abilities, as well as of measurement error, on the basis of a single underlying probabilistic model. Furthermore, the use of explanatory item response models [42, 43], which use theoretically motivated item features to predict the difficulty of individual items, can contribute to the construct validity of a test. Moreover, the problem of task impurity, found in previous approaches, can be mitigated by developing an empirically validated cognitive model of task performance via the IRT approach. Despite the theoretical and practical advantages of IRT-based testing, so far only very few established WM tasks make use of an IRT framework. Moreover, the available tests (e.g., [44]) are not available in an open computerised adaptive testing framework at present.

Using IRT as the basis of the Jack and Jill task also enables the automated generation of test items based on their item difficulties; and its implementation using Computerised Adaptive Testing (CAT, [45]). In CAT, the difficulty of items presented is matched dynamically to the current estimate of a participant’s ability. After each trial, the participant’s ability is re-estimated using the response data from the previous trial as well as the prior ability estimate. Because CAT aims to present only items that are as close as possible to the participant’s ability, it is maximally informative for estimating a participant’s ability with increasing precision. Thus, CAT helps to produce an efficient version of the test, able to capture the wide variability of WM found in heterogeneous samples, as well as mitigate fatigue effects. In particular, it offers the possibility to shorten or lengthen a task, guided by the knowledge of how changing the task’s length affects measurement error and reliability.

Our research was taken forward in multiple different settings for a few reasons. Firstly, it allowed us to recruit from a wider pool of participants, yielding a larger overall sample. Secondly, it allowed us to ensure our sample was more heterogenous and to validate our task in different cultural settings, making it more internationally robust. Thirdly, this required translating the test into several languages which are available for use in our release, and hence, makes the test more accessible. Finally, it allowed research teams to address more specific questions in separate work from that presented here.

The following sections report four studies designed to construct, calibrate, and validate a new IRT-based VSWM task that can be used in a computerised adaptive operation mode. Study 1 describes the construction and empirical calibration of the Jack and Jill task, using explanatory item response modelling of data from a large sample of the general adult population in the UK and the US. Study 2 describes the validation of the adaptive version of the Jack and Jill task in a sample of young adults from the UK and Australia. Study 3 reports the validation of the static version of the Jack and Jill task in a sample of adolescent participants from a talent development centre in Russia. Study 4 provides validation against a battery of established spatial tasks, using data from another adolescent sample from the same centre. Table 1 shows the overall design of our study.

**Table 1:** Design of the study.

	Objectives	Sample	Measures	Procedure
**Study 1**	Calibrate the static JaJ task.	244 adult participants (age range = 18–68, mean age = 31.3; SD = 10.3) recruited from the UK (32%) and the US (68%) through a market research agency.	**Static JaJ:** 14 items with increasing length from 1 to 7; two items per length.	Online test battery at home.
	Test whether it is possible to predict participants’ performance from item length.			
				~10 minutes completion time.
			Socio-demographic inventory.	
**Study 2**	Establish the validity and reliability of the static 14-item version of the JaJ task described in Study 1.	148 adult participants (age range = 18–50 mean age = 26.44; SD = 7.68) recruited through social media and on-campus advertising at Goldsmiths’ College (UK) and Macquarie University (Australia).	**Static JaJ:** Same version as Study 1.	Controlled laboratory conditions, individual quiet test cubicles.
			Backwards Digit Span [44, 46]	
			Memory Updating Figural [44, 47]	
				~15 minutes completion time.
			Socio-demographic inventory (same as Study 1).	
**Study 3**	Establish the validity and reliability of the same static 14-item version of the JaJ, used in Study 2, with a sample of adolescent high-achievers in different domains.	263 adolescent high-achieving participants (age range = 14–17, mean age = 15.45; SD = 1.00), in different domains: 112 (42.6%) in Science, 69 (26.2%) in Arts, and 82 (31.2%) in Sports at Sirius Education Centre in Russia.	**Static JaJ:** Same version as Studies 1 and 2.	Experimenter-guided, controlled conditions in groups of 10–25 people.
				~90 minutes completion time.
			Corsi block-tapping test [28]	
			The Strengths and Difficulties Questionnaire	
			[48]	
			Academic achievement (based on Russian grading system)	
			Socio-demographic inventory (different to Study 2)	
**Study 4**	Establish the validity and reliability of the adaptive JaJ (a-JaJ) with a new sample of adolescent high-achievers.	239 adolescent high-achieving adolescents (age range: 14–17, mean age = 15.09; SD = 1.02): 185 (77.4%) in Science, 9 (3.8%) in Arts and 11 in Sports (4.6%) at Sirius Education Centre in Russia.	**Computerised adaptive JaJ (a-JaJ):** Different adaptive procedure, but same item bank to Studies 1, 2 and 3.	Identical to Study 3.
	Replicate findings regarding positive association of VSWM with spatial ability and non-verbal intelligence.			
			Raven’s matrices [49]	
			Spatial ability battery [50]: Pattern assembly, Paper folding, Mechanical reasoning, Shape rotation.	
			Socio-demographic inventory (same as Study 3).	

## Study 1: Construction and calibration of the Jack and Jill task

The aim of Study 1 was to calibrate the Jack and Jill (JaJ) task. Specifically, we tested whether it is possible to predict participants’ performance from item length.

### Method

#### Participants

The sample consisted of 244 participants (41% females; age range = 18–68, mean age = 31.3; SD = 10.3). The current study utilises a simple IRT model with a constant discrimination parameter, with difficulty (item length) being the only parameter that varies across items. Thus, the current sample size is appropriately justified with respect to the guidelines for the IRT approach [41].

Participants were recruited from the UK (32%) and the US (68%). The socio-demographic background was similar across both countries: overall, 48% of participants were in part-time or full-time work, 29% were students, and 23% were unemployed and/or had small earnings from a low-income job.

#### Procedure

Participants were recruited through the Slicethepie (run by market intelligence company SoundOut, Reading, UK) online panel and forwarded to an online test battery, which comprised of study information, consent page, and the JaJ task. Participants were not instructed on what kind of device (e.g., laptop or smartphone) to use during task performance. The entire procedure took around 10 minutes to complete. The study received ethical approval from the ethics committee at Goldsmiths, University of London.

#### Materials

**Fig 1:**
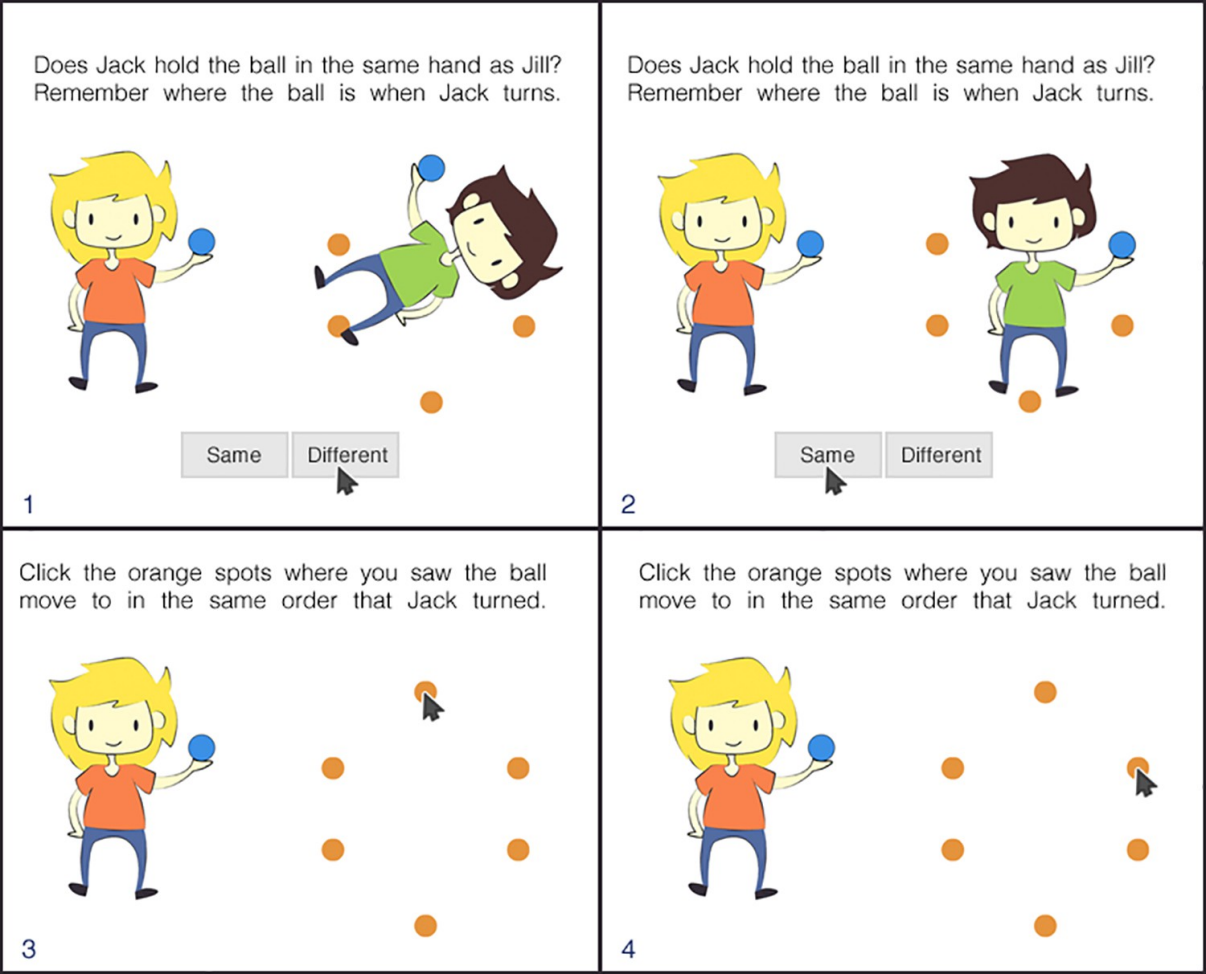
Schematic of the four consecutive screens of a JaJ trial of length two. Note: Jill always stays in the same position holding a blue ball in her right (from the participant’s perspective) hand (Panels 1–4), while Jack rotates around his axis on each stimulus presentation and can hold a ball in either his right or left hand (Panels 1–2). Jack’s ball also moves, randomly taking one of the 6 marked possible positions on the screen (orange dots). On each stimulus presentation, participants are required to perform two tasks: a) indicate whether Jack holds the ball in the same hand as Jill; and b) memorise the current ball position. At the end of each trial, participants are asked to recall the sequence of the ball positions by clicking on the marked positions in the correct order (Panels 3–4). Cursor locations represent correct answers.

*The Jack and Jill visuospatial working memory task*. The JaJ task presents participants with pictures of a young female (“Jill”) and a young male (“Jack”) on a white background (see Fig 1). The task is divided into trials of different lengths (starting with a sequence of 1 to a maximum of 7 stimuli). Each sequence length is used twice with sequence lengths increasing over time, totalling 14 trials in one testing session. For a detailed task description see Fig 1. Following the scoring procedures of the Letter Rotation task and the Mister X task, participant responses were scored as correct (1) if the whole sequence of a trial was successfully repeated or as incorrect (0) otherwise. ‘Same/different’ judgements of hand positions were not used as the main unit of scoring as this task was used only to additionally load WM processes as in a dual-task paradigm. A participant’s VSWM span is indexed by the length of the sequence of ball positions that a participant is able to recall. The JaJ task can be considered a complex span task because it requires participants to switch between memory encoding (i.e., memorising the ball’s position) and mental rotation with a subsequent decision on visual input, which creates an additional cognitive load.

A trial is considered an ‘item’ in the language of item response theory. The main parameter hypothesised to affect item difficulty is item length (i.e., the length of the sequence of ball positions).

All items were prepared in advance using independent random sequences of hand and ball positions. Ball position sequences can contain the same position more than once. No items are presented twice within one test session. In the beginning, participants are presented with step-by-step instructions and subsequently two training items of length 1 and 2 with feedback on the correctness of the responses regarding the hand judgements and ball positions. Participants do not receive feedback regarding their performance during the testing phase or after the task.

The computerised version of the JaJ VSWM task was programmed using the psychTestR [51] and Shiny frameworks [52] for the R programming language [53]. It took participants 5–15 minutes to complete.

### Results

Whilst we do not use it for formal scoring or modelling, the percentage of correct responses in the secondary “same/different” judgement task was 93.1%, suggesting the task was taken seriously and would have contributed to loading VSWM.

On the main ball position task, averaged across all 14 trials, participants demonstrated an accuracy of 35.5% correct responses (*SD* = 28.9). Male participants had an average of 33.0% (*SD* = 28.5) correct responses while females had 39.2% (*SD* = 29) responses correct. This difference did not reach the common significance level according to a Welch’s t-test (*t*(195.74) = 1.59, *p* = .11).

**Fig 2:**
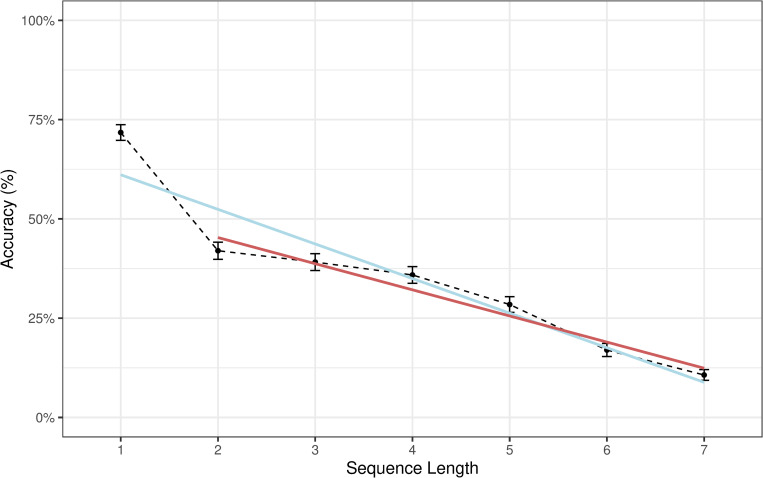
Regression lines representing the decrease in response accuracy with increasing sequence length. Note: The black line represents proportion of correct responses by length of sequences to be remembered; Error bars represent 95% CI of the proportion based on the standard error. The blue regression line was fit to sequences from length 1 to 7. The red line was only fit to sequences of length 2 to 7. The red regression line fits the empirical average accuracies connected by the dashed line fairly closely, suggesting an approximate linear trend.

After excluding items of length 1 the relationship between performance accuracy becomes approximately linear as shown in Fig 2.

In order to account for the discontinuity in performance accuracy between items of length 1 and the linear trend for items of length 2 to 7, we created a binary dummy variable that codes items of length 1 as ‘1’ and ‘0’ for all other item lengths.

To formally model participant accuracy, we constructed an explanatory item response model in the form of a binomial mixed-effects logistic regression model [42] using item length and the dummy variable for item length = 1 as fixed effects and participant ID as random intercept effect which traditionally corresponds to the participant ability parameter in IRT models. Participant responses at the item level (correct/incorrect) served as a dependent variable. The function *glmer* from the R package *lme4* [54] was employed for modelling.

According to the model, both the dummy variable and item length contributed significantly to the prediction of the correctness of the responses as shown in Table 2. As expected, longer items decreased the probability of responding correctly and an item of length 1 increased this probability. The model has a predictive accuracy of 86.7% when random effects were included. Without the random effect, prediction accuracy dropped to 71.3%.

**Table 2:** Logistic regression of item accuracy.

Term	Beta	Std. Error	z-Value	p
**(Intercept)**	0.86	0.21	4.06	< .001
**l1**	1.44	0.19	7.75	< .001
**Length**	-0.59	0.04	-16.29	< .001

Note: ‘Length’ denotes the length of the sequence of ball positions to be remembered and ‘l1’ is a binary variable indicating whether a sequence is of length = 1 or not.

In sum, the model confirmed our expectations for item length to affect task performance. In addition, the model has good predictive validity and the inclusion of individual differences information (i.e., random participant effect) explains a further considerable amount of variance in the data. Hence, the model was accepted as an explanatory model for the performance of the JaJ task. In a subsequent step, the item difficulty parameters for all item lengths as well as the discrimination parameter were extracted from this model for use with the 1-PL IRT (for technical details of parameter extraction and transformation see [43]).

Simulations using the randomCAT function from the catR package (version 3.16) [55], our derived IRT parameters and true abilities scores in range [-4, 4.15] and test lengths [5. 20] showed that the JaJ task produces scores in the range of [-1.89, 2.86]. The lowest ability value (-1.89) corresponds to the ability of remembering not more than 1 position consistently. This is a meaningful lower bound for a WM test but the assignment of the numerical value -1.89 is rather arbitrary. In the long run, it would be helpful to collect norms from a larger sample drawn from the general population (factoring in age as well), such that values of the IRT scale can be mapped to achievement percentiles in this population sample.

### Discussion

Aggregated accuracy scores show that performance on the JaJ task strongly depends on item length with the exception of items of length 1 (see Fig 2). This result may mean that items of length 1 are processed by a different mechanism, possibly visual echoic or short-term memory that does not require the engagement of the central executive and the visuospatial sketchpad, at least not to the same degree as is the case of longer items. In addition, the chance of guessing the correct response for items of length 1 is considerably higher (.167) compared to items with length > 1 (guessing level < .03). This could also have made a (small) contribution to the better performance of length 1 items. In any case, to account for the different behaviour with length 1 items, and a potential change in memory strategy, the explanatory model includes a dummy variable for items of length 1 in addition to the variable that codes item length numerically.

The item difficulty parameters and discrimination parameters extracted from this model form the basis of scoring participant ability in Studies 2 and 3 as well as the computerised adaptive version employed in Study 4. Note that by default participant ability estimates are scaled to have a mean of 0 and a standard deviation of 1 which enables the easy comparison with sample averages from subsequent studies. Item parameters of the 1-PL IRT model are given in Table A1 in the S1 Appendix.

## Study 2. Validation of the static version of the Jack and Jill task with an adult sample

Study 2 aims to establish the reliability and validity of the static 14-item version of the Jack and Jill task described in Study 1. We expect the average IRT standard error of measurement for a person’s ability to be acceptably low, indicating the task’s reliability. With regards to the validity, we expect to find significant moderate correlations between performance on the JaJ task and two established measures of WM. Moreover, we seek to conclude Study 2 with a latent variable approach. Since the construct of WM is defined as the ability to simultaneously store and actively transform information across short time spans [9, 56], VSWM can be operationalised as the ability to transiently remember and manipulate visuospatial information across time. The VSWM tasks in Study 2, Jack and Jill (JaJ), Memory Updating Figural (MUF), and Backwards Digit Span (BDS) all share the common uniting element of transiently remembering and manipulating something presented in the visual domain. Hence, we hypothesize that the common variance shared by this task set is predominantly VSWM and operationalise them as loading onto the latent variable VSWM. A good factor analysis solution should indicate the validity of this hypothesis.

### Method

#### Participants

The total sample included 148 participants (59.5% females; age range = 18–50 mean age = 26.44; *SD* = 7.68; the age and gender of 4 participants was missing, reason unknown), with a wide range of demographic backgrounds.

To assess the power of Study 2, we used the *confIntR* function from the *ufs* R package v 0.4.3 to compute the width of the confidence intervals for an effect size of .3, our sample size of 148, and a confidence level of *p* = .05 (see [57]). The resultant width was .29 [Lower bound = .15; Upper bound = .44]. In addition, we computed an achieved power with G*Power programme that turned out to be .96 [58]. This suggests that the analysis had a necessary power to detect a correlation of that size.

Participants were recruited through social media and on-campus advertising at Goldsmiths’ College, London, United Kingdom, and Macquarie University, Sydney, Australia. Participants received either course credits or a small monetary compensation for their participation. The study was approved by both the Goldsmiths’ Research Ethics Committee, and the Macquarie University Human Research Ethics Committee.

#### Procedure

Unlike in Study 1, which used uncontrolled online data collection, Study 2 was conducted under controlled laboratory conditions. All participants completed the task battery in individual quiet test cubicles. Each task had an online introduction and an example and/or training trials. A researcher was available at all times to answer questions or to help with technical difficulties. The order of tasks taken was identical for all participants: demographic questions, Backwards Digit Span, Memory Updating Figural, and JaJ.

#### Materials

*Static version of the JaJ task*. The version of the JaJ task was the same as that used in Study 1. Participants’ scores were obtained by computing weighted likelihood ability scores (θ) based on the IRT model developed in Study 1 and using the function *thetaEst* from the R package catR [59].

*Backwards Digit Span (BDS)*. BDS tasks represent a classic measure of WM [46], requiring participants to remember a sequence of digits, mentally reverse the sequence, and enter the reversed sequence. Because this BDS task required responding by clicking the numbers on a keypad, it had a particular visuospatial quality to it compared to other BDS tasks. It was a re-implementation of the BDS used by Vock and Holling [44] and consisted of 12 trials of increasing difficulty using sequences with four to seven digits. The original IRT models for this task [44] could not be used for scoring as they also included items from a different WM paradigm in their modelling. Therefore, scores were computed for each participant a) by simply summing correct responses and b) by modelling the data with a 1-PL IRT model, which then served to compute ability scores using the weighted likelihood method [60]. Sum scores and IRT ability scores (which are non-independent) were very highly correlated (*r* = .99). IRT ability scores were used for further analysis. Since all stimuli were presented in the visual domain and responding involved clicking digits on a visually displayed keypad which spatially organised the digits, we consider this a visuospatial BDS task.

*Memory Updating Figural (MUF)*. The MUF task is a visuospatial task similar to the task used in Salthouse, Babcock, and Shaw [47] and is also a re-implementation of a task designed by Vock and Holling [44]. Participants were presented a variable number of rectangles where dots could appear in any corner for 1,500 ms at a time, followed by arrows pointing to other corners of the same rectangles. Participants had to click where the dots would end up after mentally applying the arrow transformations. The MUF comprised 14 items which increased in difficulty based on the number of mental operations to be completed. The original IRT models for this task [44] could not be used for scoring, as they also included items from a different WM paradigm. Similar to the BDS task, participants’ sum scores and weighted likelihood IRT scores (which are non-independent) were very highly correlated (*r* = .98), and IRT ability scores were used for the subsequent analysis.

### Results

Because the gender of 4 participants was missing (reason unknown), an independent samples t-test using pairwise deletion (resulting in 134 cases with complete test scores) was conducted and did not indicate any gender differences (mean θ_male_ = 0.68, SD θ_male_ = 0.95, mean θ_female_ = 0.61, *SD* θ_female_ = 0.98, *t* = -0.42, *p* = .68). Further analysis was carried out on the whole sample. Descriptive statistics for the whole sample are presented in Table 3.

**Table 3:** Descriptive statistics for all performance tasks in Study 2.

Variable	N	Sum Score Mean (SD)	IRT Score (θ) Mean (SD)	Range	Kurtosis	Skewness
Age	144	26.44 (7.68)		18–50	.15	.97
BDS	147	5.88 (3.07)	-.01 (1.13)	-2.79–2.73	.29	-.18
MUF	147	6.37 (3.81)	.15 (.91)	-2.01–2.93	.19	-.03
JaJ	142	7.36 (4.30)	.63 (.97)	-1.50–2.36	-.48	-.36

Note: BDS—Backwards Digit Span; MUF—Memory Updating Figural; JaJ–Jack and Jill task.

Participant IRT ability scores (θ) on the JaJ task ranged [-1.5, 2.36] with a mean of 0.64 (SD = 0.97); the theoretical and empirical range of the sum scores was BDS [0… 12], MUF [0… 14], JaJ [0… 14]. This indicates that the sample of participants tested under laboratory conditions in Study 2 had a higher average but a very similar standard deviation of θ-scores compared to the calibration sample from Study 1, where the mean θ-score was 0 with a standard deviation of 1. Using the explanatory IRT model to convert the θ-scores back to the standard scale of the VSWM tasks indicated that participants in Study 2 had a mean complex memory span of 4.01 (*SD* = 2.05). However, note that the prediction of complex WM span ability values from IRT θ-scores can produce WM span estimates outside the scale of the task (i.e., < 0 and > 7). Therefore, only 82.7% of participants with a converted WM span estimate between 0 and 7 (after rounding) were included in the calculation of the mean complex memory span. 5% of participants had an estimated score below and 12.2% an estimated score above this range. For comparison, the mean θ-score of 0 in the calibration sample corresponds to a complex span estimate of 2.4.

The average standard error of measurement (SEM) of the θ-scores was .37. The marginal empirical reliability computed from the standard errors of measurement was .83. As expected, all three WM task scores correlated positively and significantly (p-values < .001 after correcting for multiple comparisons using Holm’s procedure [61]). The JaJ had moderate correlations with both the MUF task (*r* = .44) and the BDS task (*r* = .37) (see Table 4).

**Table 4:** Pairwise correlations of all tasks.

	BDS	MUF	JaJ
**BDS**		.38***	.37***
**MUF**			.44***
**JaJ**			

Significance is denoted as

* = *p* < .05

** = *p* < .01

*** = *p* < .001.

We proceeded to assess the hypothesised measurement model for the VSWM factor with a minimum residual exploratory factor analysis. All factor loadings were > .50 which indicated that the tasks represented the factor well (see Table 5). The VSWM latent variable explained 47% of the variance in the observed VSWM task. JaJ had a uniqueness value (u^2^) of .66.

**Table 5:** Exploratory factor analysis results for the hypothesized VSWM factor.

VSWM			
Task	Factor loading	h^2^	u^2^
**BDS**	.66	.43	.57
**MUF**	.80	.64	.36
**JaJ**	.58	.34	.66

### Discussion

The data from Study 2 shows that the JaJ task moderately correlates with two other tasks purported to measure aspects of (VS)WM capacity (backwards digit span and a visuospatial memory updating task), suggesting that it is a valid measure of an aspect of WM. The good factor analysis solution suggests that the task set combines well to reflect a latent variable, which was hypothesized to indicate VSWM. Whether or not VSWM is the underlying latent variable, the factor analysis shows there is a latent factor among the three tasks, which is probably more specific than *g*, since three tasks involve manipulating visually presented information, and is therefore likely to do with VSWM. However, this represents an assumption, rather than a proof.

However, JaJ was correlated more strongly with the visuospatial updating task (measured by MUF; *r* = .44) than with the memory span task (measured with the BDS; *r* = .37) in the same sample of participants. This may mean that the JaJ task taps into the visuospatial component of WM more than verbal or numerical components and could serve as a task to assess the capacity of the visuospatial sketchpad construct. Hence, these correlations could also suggest the divergent validity of the newly developed measure within WM modelling. However, the *R* package *cocor* v1.1–3 [62] indicated that the correlations were not statistically different from one another, according to Steiger’s [63] procedure (*z* = 0.94, *p* = 0.35).

Taken together, the factor analysis suggests the task set indicates shared variance to do with similar VSWM processes (e.g., short-term visual memory/manipulation). Yet, the moderate sizes of correlations and the presence of substantial unexplained variance in the factor solution suggest the three tasks might tap into slightly different constructs within WM more generally (e.g., MUF and BDS measure aspects other general aspects of WM capacity and not specifically VSWM).

The average mean standard error of measurement for the JaJ task was .37, i.e., about 1/3 of a standard deviation of the ability scores, which can be considered acceptably low. The marginal reliability of .83, as computed using the marginal_rxx() function from the R package mirt, was in a good range. This measure of reliability in an IRT-context corresponds to Cronbach’s α from CTT [64, 65]. However, a limitation of our approach is that we do not know the test-retest reliability of our derived score.

Average WM ability estimate, and the corresponding complex span capacity, were considerably higher in Study 2 compared to Study 1. This ability to discriminate between the two samples (Study 1 vs. Study 2.) can be considered as an additional strength of the JaJ task. Several factors may contribute to these differences. First, the testing conditions were different: controlled laboratory setting in Study 2 vs. uncontrolled online conditions in Study 1. Second, different recruitment criteria were applied: predominantly student population with an age cap at 50 in Study 2 (to focus on the demographic our sample of convenience) vs. participants from a market research panel representing the general population without any age cap in Study 1. This result is consistent with previous evidence showing that WM performance differs as a function of college status (e.g., [66]). Besides, because participants in Study 1 completed the study outside of the laboratory, at home, they could have used different devices (e.g., laptops, tablets, or mobile phones), which could also affect accuracy. Finally, a future study should specifically target construct validity with a measure of fluid intelligence and other WM tasks included alongside the JAJ to explore the net of nomothetical relationships of the JAJ more closely. Although, we note that measures of fluid intelligence and WM tend to exhibit reasonably sized correlations.

## Study 3. Validation of the static version of the JaJ task with high-achieving adolescents

Study 3 was designed to establish the validity and reliability of the same static 14-item version of the JaJ, as used in Study 2, in a sample of adolescent high-achievers in different domains. In terms of the test’s validity, we aimed to replicate findings from the literature regarding associations of VSWM with socio-demographic variables, other tests of VSWM, academic achievement, socio-emotional skills, and the type of domain expertise (e.g., Science vs. Arts). Specifically, we hypothesised that performance on the JaJ task is positively associated with adolescents’ age [23, 66] but not with gender [67]. Moreover, performance on the JaJ task should be substantially correlated with the performance on the Corsi Block-tapping test (CBTT, [34])–a well-established measure of VSWM; as well as with measures of academic achievement [68]. In contrast, we did not expect to find any substantial correlations with a socio-emotional measure of behavioural difficulties (Strength and Difficulties questionnaire, [48]) because of the lack of theoretical or empirical evidence for strong relationships between VSWM capacity and socio-emotional traits in non-clinical populations. Finally, previous research suggests that individuals with different expertise vary in the aspects of VSWM capacity. For example, musical training was associated with visuospatial sequence learning advantage [69]; sports training was associated with superior visuospatial attention and memory performance [70, 71]; success in natural sciences was related to the overall visuospatial processing [21, 72]. Thus, we expect that participants with expertise in different domains should show differences in JaJ scores.

### Method

#### Participants

The participants (*N* = 263; 47.3% females, 50% males, 7 participants did not provide information on gender; age range = 14–17, mean age = 15.45; *SD* = 1.00) were recruited at the educational centre Sirius in Sochi, Russia. The centre supports high-achieving adolescents from different regions of Russia by providing them with intensive educational and training programmes. To be invited for a 1-month educational programme in the centre, adolescents (10 to 17 years of age) are required to show high performance in school subjects (biology, chemistry, mathematics, physics, etc.) and subject competitions/Olympiads; high performance in Sports (hockey, figure skating, and chess); or Arts (performing arts, painting, ballet, literature). The participants represented three different educational tracks, with 112 (42.6%) recruited from the Science, 69 (26.2%) from Arts, and 82 (31.2%) from Sports tracks. None of the participants was excluded. More detailed sample description is available in Tsigeman [73].

#### Procedure

The participants and their parents or guardians received information regarding the goals and procedures of the study and the voluntary basis of their participation. Only the students whose parents or legal guardians provided written consent participated in the study. Additionally, assent was obtained from the adolescent participants before the testing session. Participants filled in questionnaires and performed cognitive tests under similar controlled conditions in groups of 10–25 people. Group testing was employed to fit a tight schedule of adolescents’ the intensive educational programme that did not have specific scheduled time for cognitive testing. The testing session lasted about 90-minutes and the order of tasks was the same across the groups. An experimenter guided all testing sessions. All the tasks had written instructions that were repeated verbally by the experimenter. Participants did not receive any compensation for their participation or any feedback on their performance. The study was approved by the Ethics committee of the Interdisciplinary Research at Tomsk State University (code of ethical approval: 16012018–5).

#### Materials

*Static version of the Jack and Jill task*. The same 14-item static version of Jack and Jill task as in Studies 1 and 2 was administered. Two independent translators whose first language is Russian and the second English conducted the translation of the test from English to Russian following the ITC guidelines for test translations [74]. For assessing reliability, we computed reliability measures using classical test theory as well as IRT.

*Corsi block-tapping test*. To assess the participants’ VSWM span, using an established test [75, 76], a computerised version of Corsi block-tapping test (CBTT) was used. Only forward version of the Corsi task was employed as multiple studies found no difference between forward and backward recall procedures [77–80]. CBTT usually shows satisfactory reliability estimated by Cronbach’s α = .79 [28] and split-half reliability = .86 [81]. The task was presented through the Pebl 2.0.11 software [82]. Participants were presented with nine irregularly arranged blue blocks on a black background. Each trial started with blocks lighting up in yellow in particular sequences of increasing length from two to nine blocks (all the sequences were generated by Kessels and collaborators [76]). Each sequence length (2–9) was presented twice, adding up to 16 trials with three training trials in the beginning. There was a 1000ms inter-stimulus interval. After presentation, participants had to reproduce the sequence in the same order using a computer mouse. The next trial started immediately after the previous sequence had been reproduced and the participant pressed the button ‘done’. The test terminated if the participant did not correctly recall the sequence of the current length twice consecutively. A sequence was scored as correct if the participant was able to reproduce the entire sequence without error. The final scores were calculated as a number of correctly recalled trials.

*Academic achievement*. The participants reported their last year Russian language and algebra school grades which varied from 1 to 5 according to a Russian grading system. As students with unsatisfactory marks (1 and 2) cannot be selected to attend Sirius, the score distributions for both grades were restricted in the current sample.

*The strengths and difficulties questionnaire*. The Strengths and Difficulties Questionnaire (SDQ) assesses behavioural adjustment and behavioural difficulties in children and adolescents (4–17 years). It consists of five subscales: emotional problems, peer problems, conduct problems, hyperactivity, and prosocial behaviour. The questionnaire asks participants about their social relationships, anxiety symptoms, and behavioural difficulties. Participants answer each question using a 3-point Likert scale where 0 = ‘not true’, 1 = ‘somewhat true’, and 2 = ‘certainly true’. We used the Russian 25-item version (freely available at https://www.sdqinfo.org/a0.html). We only employed the overall behavioural difficulties score here, obtained as a sum of all sub-scales except prosocial. The reliability of the total behavioural difficulties in the English version of the questionnaire equals .80 as measured by Cronbach’s α [83]. The Russian version showed a Cronbach’s α equal to .65 for total behavioural difficulties in a sample of adolescents [84, 85].

### Results

Descriptive statistics for all measures are presented in Table 6. Skewness and kurtosis of most variables except for JaJ θ varied within an acceptable range (i.e., below the cut-off of 2 as recommended by Field [86]). The relatively high values for kurtosis and skewness of the JaJ sample distribution are expected for this selected sample, which included almost 50% of adolescents, showing high achievement in STEM (Science, Technology, Engineering, and Mathematics). STEM-selected adolescents were previously shown and assumed to demonstrate extremely high VSWM capacity [14, 20, 21].

**Table 6:** Descriptive statistics for variables used for analysis in Study 3.

Variable	N*	Mean (SD)	Range	Kurtosis	Skewness
Age	262	15.45 (1.00)	14–18	-.63	-.19
JaJ θ	262	.94 (.68)	-1.50–2.36	2.73	-1.29
CBTT	218	9.11 (1.61)	5–13	-.11	.21
Total behavioural difficulties	214	9.50 (3.90)	2–26	-.50	.37
Russian language grade	262	4.48 (.61)	3–5	-.75	-.70
Algebra grade	261	4.52 (.61)	3–5	-.12	-.92

Note: JaJ–Jack and Jill working memory task, CBTT–Corsi block-tapping test

*different N for tests occurred because not all participants managed to perform all tasks with in the 1.5h session.

JaJ θ scores ranged [-1.50, 2.36] with a mean of .94 (*SD* = .68), corresponding to an average complex WM span of 4.99 (*SD* = 1.63). This score was computed from 90.1% of the participants with a converted complex span score between 0 and 7 (0.8% scoring < 0 and 8.5% scoring > 7). Thus, the high-achieving adolescents in Study 3 were able to remember one extra position on average, compared with adults in Study 2.

In addition, JaJ θ scores had significant positive associations with all measures of academic achievement and the CBTT as shown in Table 7. In contrast, the correlations between JaJ θ scores and self-reported behavioural difficulties were non-significant at the .05 level.

**Table 7:** Pearson’s correlations between behaviour, achievement and VSWM measures.

	1	2	3	4	5
1 JaJ θ	-				
2 CBTT	.28***	-			
3 Total behavioural difficulties	-.11	-.09	-		
4 Russian language grade	.15*	.01	-.05	-	
5 Algebra grade	.31***	.11	-.03	.53***	-

Note:

*p < .05

**p ≤ .01

***p ≤ .001; JaJ–Jack and Jill working memory task.

The focus of the analysis was on correlations which were shown to stabilise at a sample size of 250 [87]. A *post hoc* analysis of achieved power showed that with *N* = 263, a weak-to-moderate effect size of ρ = [0.15–0.53] and the conservative parameters of α = .01 and a two-tailed hypothesis for a bivariate normal correlation model suggested that achieved power was in a range [0.45, 0.99].

**Fig 3:**
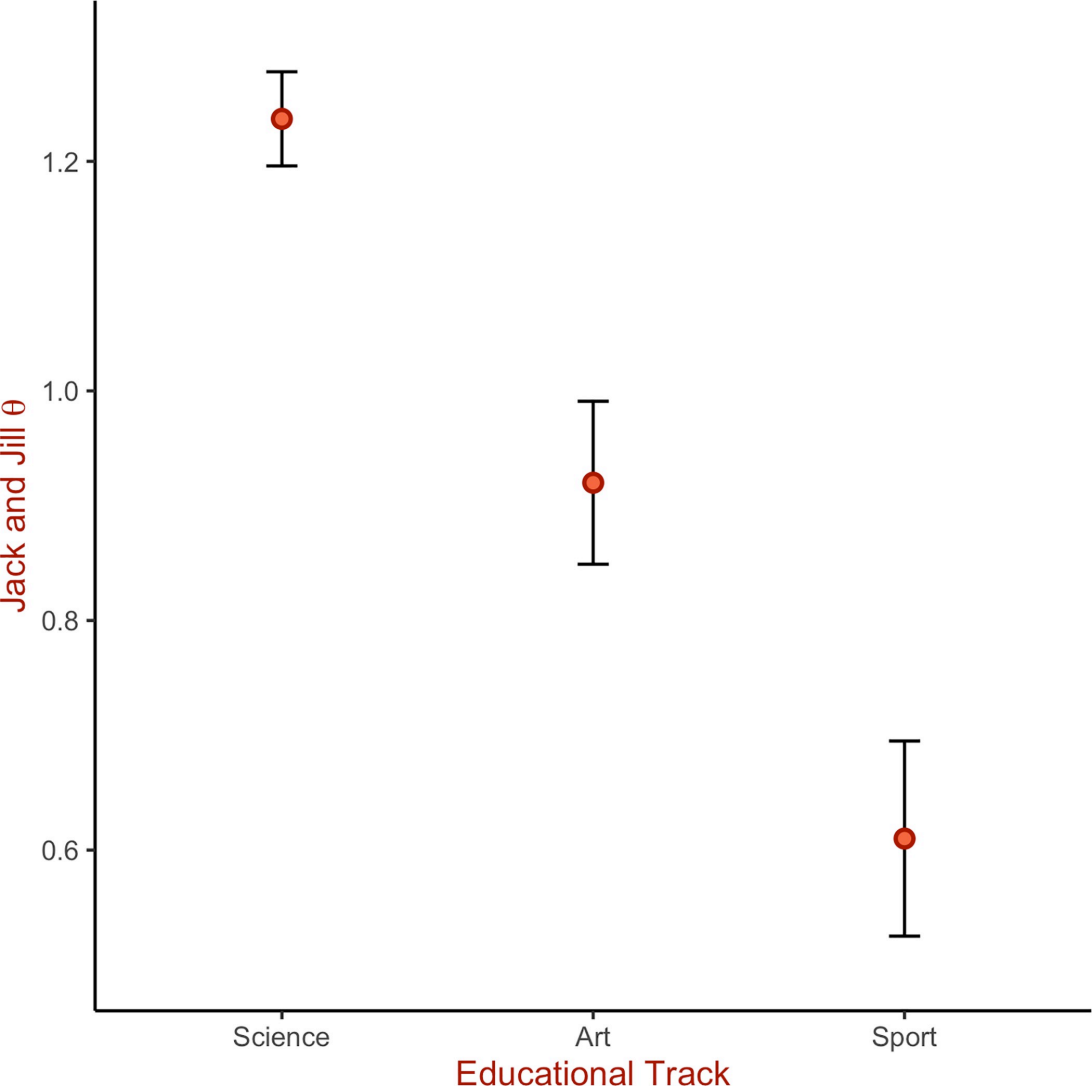
Jack and Jill task performance by educational track. Note: Error bars represent the 95% CI around the mean.

An independent samples t-test suggested equal performance of males (mean θ = .96, *SD* = .74) and females (mean θ = .91, *SD* = .62; *t* = .60, *p* = .54). JaJ θ scores showed a significant positive correlation with the age of participants (*r* = .19, *p* < .003). However, the effect of age is confounded by age differences across the three educational tracks. Science (mean age = 15.77, *SD* = .68) and art (mean age = 15.83, *SD* = 1.07) students were significantly older (*F*(2, 259) = 44.43, *p* < .001) than sport students (mean age = 14.69, *SD* = .91). A linear model with age and educational track as independent variables and θ as dependent variable showed that the main effect of age and the interaction between age and track were non-significant (*p* = .30 and *p* = .56, respectively) while the main effect of track was significant (*F*(2, 246) = 5.16, *p* = .006, η_p_^2^ = .04). Post-hoc tests showed that participants from the science track had significantly higher JaJ scores (mean θ_Science_ = 1.23, SD = .44) in comparison to art (mean θ_Art_ = .84, SD = .70) or sport students (mean θ_Sports_ = .61, SD = .77), while art students outperformed sport students (see Fig 3).

Measures of reliability and internal consistency from classical test theory as well as IRT indicated that the JaJ task was sufficiently reliable by common benchmarks. The average standard error of measurement for the IRT-based theta scores was sufficiently low (SEM(θ) = .33). Cronbach’s α for the 14-item static version of the test was .78 and the equivalent empirical marginal reliability derived from the underlying IRT model was .79.

### Discussion

The aim of Study 3 was to assess the validity and reliability of the JaJ VSWM task in a sample of adolescents selected for high achievement in Science, Arts, or Sports. The test showed sufficient reliability as shown by indicators from classical test theory and IRT. JaJ also showed good concurrent validity as evidenced by the correlation with Corsi Block-tapping test score. The moderate correlation (*r* = .28) with the CBTT was in a similar range to the correlation (*r* = .36) between letter rotation scores and CBTT reported by Miyake and collaborators [34]. The moderate correlation between the two tasks may be explained by the fact that JaJ uses a dual-task paradigm, which requires both information processing from the central executive and information storage in the visuospatial sketchpad component, whereas the CBTT only requires encoding and retrieval from visuospatial memory and is not based on a dual-task paradigm.

Furthermore, the data showed a significant and positive association between performance on the JaJ task and age, which disappeared after controlling for the track. The absence of a reliable association between JaJ task performance and age might be due to the narrow age range (14–18) but is consistent with previous literature [23, 66]. In addition, we found correlations of small to moderate size between JaJ scores and measures of academic achievement: Algebra and Russian language. This is consistent with previous research which showed VSWM was related to both maths [14, 88] and reading abilities [14, 18]. JaJ performance explained a small to moderate amount of the variance in the grades data (from 2% in the Russian language to almost 10% in Algebra grades). The stronger associations with maths compared to language grades are in line with previous studies [14, 67]. It is possible that VSWM specifically helps to represent number magnitudes spatially in the form of a mental number line and thus may help to represent maths tasks schematically and solve them efficiently (e.g., [89]). A moderate correlation between JaJ performance and maths achievement is consistent with the correlations of .31 between VSWM and maths reported in the recent meta-analysis by Peng, Namkung, Barnes, and Sun [90]. A correlation between Russian language school grade and JaJ task is lower than the correlation between English achievement and VSWM measures (.29 and .33) reported in Jarvis and Gathercole [91] and a similar correlation between WM and reading abilities of *r* = .29 reported in the meta-analysis by Peng and collaborators [92]. Overall low correlations between JaJ score and school achievement could be explained in the light of limitations of the achievement measures: the restricted scale [3… 5], restricted variance in this selected population (54% of people with the highest grade on Russian language and 59% of people with the highest grade on Maths) and potential biases and differences in standards, reflected in the teacher ratings [84, 85, 93, 94]).

As in Studies 1 and 2, no significant gender differences were found for JaJ scores in Study 3. This is at odds with some previous studies (e.g., [95]) which report male advantage in VSWM tasks performance. However, numerous other studies similarly report non-significant gender differences (e.g., [66, 96]). Gender differences obtained in some studies may be due to the modality of testing or the parameters measured, reflecting the use of different strategies [97] or differences in other abilities such as spatial [98, 99]. Therefore, the JaJ task can be considered a non-biased test with respect to gender.

The results from Study 3 showed that, on average, STEM-selected students significantly outperformed Arts and Sports students, while Arts students outperformed Sports students. These differences in VSWM may arise from the different expertise profiles of the participants as suggested by previous literature [67, 69, 70]. However, the large variability in the VSWM abilities within the Arts and Sports tracks is worth noting and may be explained by the fact that participants from the Arts track comprised of ballet dancers, musicians, and visual artists, while participants from the Sports track included hockey players, figure skaters, and chess players. Thus, the heterogeneity of expertise within the tracks might have influenced differences in VSWM between tracks. Further research is needed to evaluate the JaJ task performance in specific sub-domains of expertise.

## Study 4. Implementation and validation of the computerised adaptive version of the JaJ task with high-achieving adolescents

A limitation of WM tasks that are not adaptive is that they can be time-consuming and perceived as tedious by participants. This is because static tests present items that are either too difficult or too easy for an individual. Computerised adaptive testing reduces the test completion time and minimises fatigue, which potentially increases the reliability of the test. For an in-depth discussion about more of the merits of adaptive testing, we refer the reader to Harrison & Müllensiefen [100].

In Study 4, we implemented a computerised adaptive version of the JaJ task (a-JaJ) based upon the IRT model described in Study 1. Study 4 was designed to establish the validity and reliability of the a-JaJ with a new sample of adolescent high-achievers.

We also aimed to replicate findings from the literature regarding the positive association of VSWM with measures of spatial ability [34] and non-verbal intelligence [12, 101]. In accordance with Study 3, we expect a positive association between performance on the a-JaJ and different academic subjects, but not with gender and age. For assessing the reliability of the a-JaJ, we computed reliability measures based on the underlying IRT model.

### Method

#### Participants

Similar to Studies 2 and 3, Study 4 utilised bivariate correlations as a methodology, and the same rationale presented earlier suggests that its sample size (*N* = 239 high-achieving adolescents; 31% females; 52.3% males, 40 participants did not provide information on gender; age range: 14–17, mean age = 15.09; SD = 1.02, 35 participants did not provide information on age) was reasonable to find a moderate effect size. Adolescents were recruited at the educational centre Sirius (see Study 3 for details) from three tracks: Science (185, 77.4%), Arts (9, 3.8%) and Sports (11, 4.6%), and 34 participants did not provide information on educational track. No participants were excluded.

#### Procedure

Procedures were identical to Study 3.

#### Materials

Participants filled in the same computerised socio-demographic inventory as in the Study 3.

*Computerised adaptive version of the JaJ task*. In addition to the programming dependencies listed earlier, the a-JaJ utilised the *psychTestRCAT* [51] and *catR* [55, 59,] packages for R. The a-JaJ employs a precalculated item bank with randomly generated items of lengths [1… 7].

The first item administered in each test session is constrained to be a random item of length 2, corresponding to an average ability level of 0.13 on the z-score metric of the IRT model. After responding to an item (i.e., a trial of ball sequences) the participant’s ability is re-estimated using Bayes modal estimation with a Gaussian prior of mean 0 and standard deviation 1 and using the item difficulties from the IRT model described in Study 1 (for additional information see Table A1 in S1 Appendix). The next item is then randomly selected from the subset of items in the item bank that are maximally close in difficulty to the current ability estimate using Urry’s criterion [59]. As in Studies 1–3, the test was terminated after 14 items. Additionally, at the end, the new adaptive test version recomputes the participant ability estimate using weighted maximum-likelihood estimation [60].

*Raven’s matrices*. Raven’s progressive matrices [49] were used to assess the level of non-verbal intelligence of participants. The test included a set of 30 matrices of increasing difficulty, each of which contains a missing element. Participants choose the missing part out of 6 or 8 possible variants. Elementary sets (A, B) from the original set were dropped from the test. Items 1, 3, 5, 7, 9, 11 from parts C to E and all 12 items from part F were selected to cover the expected high ability range of the participants.

*Spatial ability battery*. Four tasks from the computerised spatial ability battery King’s challenge [50] (for Russian adaptation see [102, 103]) were used (See Table 8 for the tasks descriptions). A subtest is ended once a participant gives incorrect answers on four consecutive items.

**Table 8:** Spatial ability tasks.

Task name	Task	N of items	Time limit per item (sec)
Pattern assembly	Decide how a final figure shape will look considering that stimuli elements are attached to each other in a specified way	15	20
Paper folding	Decide where the holes will appear if a sheet of paper was folded in a specific way and then pierced and unfolded	15	20
Mechanical reasoning	Explain the functions of different mechanisms (shafts, gears, etc.)	16	25
Shape rotation	Select a figure among several alternatives which is identical to a target figure presented from a different angle	15	20

### Results

Descriptive statistics for all measures are presented in Table 9. Skewness and kurtosis of most variables except for a-JaJ θ varied with an acceptable range (i.e., below the cut-off of 2 as recommended by Field [86]). Again, the relatively high values for kurtosis and skewness are presumably due to the selected sample used in this study.

**Table 9:** Descriptive statistics for study variables.

variable	N*	Mean (*SD*)	Range	Kurtosis	Skewness
Age	204	15.09 (1.00)	14–18	-.94	.46
a-JaJ θ	234	1.16 (.54)	-.80–2.74	3.81	-1.28
Raven’s score	127	19.65 (4.90)	3–29	.03	-.48
Pattern assembly	116	7.41 (2.76)	1–12	-.42	-.51
Paper folding	107	9.82 (9.82)	0–15	-.09	-.90
Mechanical reasoning	105	10.70 (2.81)	3–16	-.45	-.44
Shape rotation	110	9.21 (3.78)	1–15	-.73	-.57
Russian language grade	205	4.58 (.59)	3–5	.15	-1.07
Algebra grade	202	4.69 (.56)	3–5	1.71	-1.63

Note: a-JaJ–computerised adaptive Jack and Jill working memory task

*different N for tests occurred because not all participants managed to perform all tasks in 1.5h session.

a-JaJ θ scores varied from -.80 to 2.74 in the current sample, with a mean of 1.16 (*SD* = .54). This corresponds to an average WM complex span capacity of 5.34 positions (*SD* = 1.56) computed from the scores of those 77.7% of participants with a score between 0 and 7. Only 2.1% of the participants had an estimated complex span below 0, but 22.2% of participants had estimated span scores > 7. As expected, a-JaJ θ had significant positive associations with all measures of academic achievement (Table 10). It did not show a significant correlation with the age of participants (*r* = -.01, *p* = .80) or reveal any significant difference of the JaJ performance (*t* = .93, *p* = .33) for females (mean θ = 1.09, SD = .55) and males (mean θ = 1.17, SD = .62). JaJ was quite strongly correlated with the Raven’s matrices task (*r* = .57) and the non-WM visuospatial measures (*r* = .46-.54).

**Table 10:** Pearson’s correlations between JaJ, general cognitive ability, spatial abilities and achievement measures.

	1	2	3	4	5	6	7	8	9	10
1 JaJ θ	-									
2 Raven’s score	.57***	-								
3 Pattern assembly	.46***	.46***	-							
4 Paper folding	.54***	.66***	.60***	-						
5 Mechanical reasoning	.51***	.54***	.59***	.67***	-					
6 Shape rotation	.52***	.66***	.59***	.79***	.56***	-				
7 Russian language	.15*	.07	-.00	.10	-.03	.06	-			
8 Algebra	.21**	.31**	.22*	.34**	.32**	.23*	.61***	-		

Note:

*p < .05

**p ≤ .01

***p ≤ .001; JaJ–Jack and Jill working memory task.

**Fig 4:**
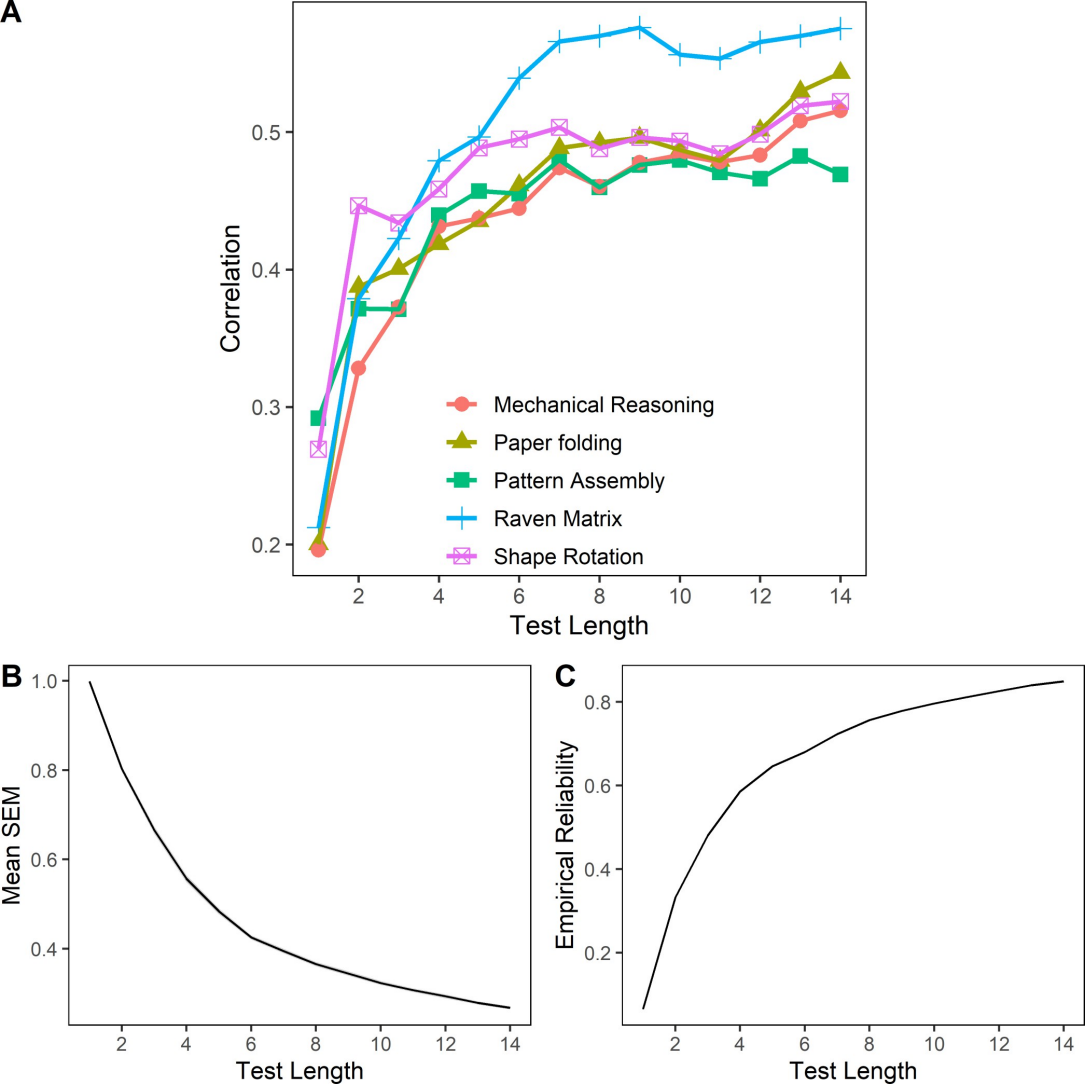
Indicators of correlational test validity, measurement error and reliability by task length. A. Correlations with other cognitive performance tasks by length of the a-JaJ test. B. Standard measurement error by test length. C. Marginal reliability of test scores by test length.

Fig 4 (Panel A) expands this correlational information for the a-JaJ task at different lengths, (i.e., as if the task had been terminated after 1, 2, 3, …, 14 trials). The figure shows how correlations with the different spatial ability subtests change as the number of trials on the JaJ task increases. For most spatial ability subtests as well as for Raven’s matrices score correlations seem to reach a plateau at around 7 trials, after which the value of the correlation coefficient changes only to a considerably smaller degree.

Both indicators of reliability (i.e., the mean standard error of measurement (SEM) of the IRT θ scores and the empirical marginal reliability of the scores) suggest that the a-JaJ task after 14 trials has a good level of reliability. Mean SEM was .27 and hence about a quarter of a standard deviation of the θ scores variability. The corresponding empirical marginal reliability was .80. Fig 4 (Panels B and C) shows how measurement error decreases and reliability of the test increases as the number of trials of the a-JaJ grows. In contrast to the correlations with the spatial ability battery in Fig 4 (Panel A), reliability does not seem to reach a plateau. However, the gain in reliability diminishes gradually as the number of trials increase.

### Discussion

The aim of Study 4 was to test the validity and reliability of the adaptive JaJ with a sample of adolescents selected for high achievement. After 14 trials, the a-JaJ had an acceptably low measurement error and showed good reliability. These benchmark indicators were slightly better for the 14-item adaptive version in Study 4 compared to the 14-item static version used in Study 3. The decreased measurement error and slightly larger reliability can be explained by the fact that, for each trial, the adaptive mechanism (described in the Method section) selects the item that maximises the information gathered about an individual participant’s ability. However, the small gain in reliability is only a secondary benefit of the adaptive version. The primary benefit of adaptive IRT-based tasks is the possibility to shorten or lengthen the task guided by the knowledge of how changing the task’s length affects measurement error and reliability. As expected, the task’s reliability increases with the number of trials as depicted in Fig 4 (Panel B and C). The information from these figures provides the basis for principled decisions in practical application scenarios where overall test time is limited and when the trade-off between test length and measurement error needs to be considered. The a-JaJ also demonstrated some concurrent validity through the significant and substantial positive correlations with the spatial ability tasks and the non-verbal intelligence task. The correlations with spatial ability measures varied from .46 to .54, which is similar to the correlations between VSWM measures and spatial visualisation measures (*r* = .42 –.49) reported in Miyake and collaborators [34] and in Kane and collaborators (*r* = .39 –.51) [104]. This suggests that the a-JaJ also partially taps into spatial ability (specifically, spatial visualisation) and non-verbal intelligence.

Furthermore, the data of Study 4 replicated the results of Study 3 regarding substantial associations between VSWM performance and academic achievement measures. Lower correlations between a-JaJ scores and achievement measures might be due to narrower variance in variables in comparison to Study 3. This is because the sample in Study 4 consisted of mostly Science students (77.4%) who received high school grades and performed JaJ at a very high level. The data from Study 4 again show no gender and age differences for the task. It is also worth noting that more than 20% of the participants demonstrated estimated span scores > 7, which suggests a possible ceiling effect for this sample. Further research is needed to adapt the task for high-ability individuals.

## General discussion

The current study aimed to construct, calibrate and validate a new IRT-based computerised adaptive VSWM task. It had the objective to create a VSWM task which is a) suitable for both adult and adolescent populations; b) based on an explanatory IRT model, contributing to the construct validity and flexible use of the task; c) implemented using CAT and enabling automated generation of test items based on their item difficulties.

In a first step, a new dual-paradigm VSWM task called Jack and Jill was constructed and calibrated using explanatory item response modelling based on data from a sample of the general UK adult population. Then, the static version of the task was assessed for its validity and reliability in a sample of adults from the UK and Australia and a sample of high-achieving adolescents from Russia. Finally, the computerised adaptive version of the JaJ (a-JaJ), was developed and validated with a second sample of high-achieving Russian adolescents.

Across all studies, the JaJ task showed sufficient internal consistency and reliability as indicated by a low standard error of measurement and acceptable to good values of empirical marginal reliability. It also demonstrated sufficient concurrent validity, (e.g., by substantial correlations with all spatial ability measures and the non-verbal intelligence measure in Study 4) and convergent validity, as indicated by significant correlations with other established measures of WM capacity (Backwards Digit Span, Memory Updating Figural, Corsi Block-tapping test), spatial tasks (Paper Folding, Shape Rotation, Mechanical Reasoning, Pattern Assembly) and a non-verbal intelligence task (Raven’s progressive matrices). Interestingly, we found larger correlations for JaJ with intelligence and SA (*r* = .57 with Raven’s matrices, .51–.54 with spatial ability measures) compared to its correlations with other VSWM measures (.37 with BDS, .44 with MUF, .28 with CBTT). However, no study included all the aforementioned measures, and thus differences in the magnitude of these correlations, cannot be directly compared. If true, these differences could be due to several reasons, including: a) the difference in sample sizes in the studies (*N* = 263 and *N* = 148 respectively), b) effect sizes in Study 3 could be inflated due to a restricted range effect, very similar to the situation described in the appropriate section of Coe [105]; comparison to the general population should be treated with caution, c) Study 2 scored higher on measures of reliability which may yield a more representative, but realistically smaller, effect size, and d) whilst the BDS task is visually presented, it is perhaps more related to general components of WM rather than being a pure measure of VSWM.

It was important in the context of the four studies to limit the duration of the JaJ (as we chose to be 14 items, 2 of each length 1 to 7) because in each study it was part of a larger battery of tests. However, the underlying adaptive functionality derived in Study 4 allows users to specify an error threshold criterion for terminating the test (at the expense of an a priori unknown test duration) and hence flexible adjustment of the task’s overall duration, (e.g., to be compatible with the constraints of the testing context). The results from Study 4 suggest that reliability and validity of the task are acceptable after 7 or 8 trials (5–6 minutes), with diminishing gains from more trials.

The a-JaJ mean scores for high-achieving adolescents in Study 4 were substantially higher than for the samples from Studies 1 and 2 but similar to the sample of Study 3. The fact that, on average, high-achieving adolescents scored substantially higher than adult participants from the general population and young adults (predominantly students) suggests that the task could be an element in a battery for identifying high cognitive potential or talent. This is in line with research showing the importance of working memory capacity for high achievement in maths, music, and visual art (e.g., [106]). No gender differences were found in any of the four studies.

There are also some limitations to the current version of the JaJ. The current version of the task contains only items up to length 7 which generated scores distributions that were slightly left-skewed with a heavy left tail for the samples of high-achieving adolescents in Studies 3 and 4. Note that only these latter two studies included a substantial set of additional measures. Hence, we cannot generalise the findings regarding the pattern of correlations beyond the population of high achieving adolescents.

An extended version of the JaJ task should include an item bank with longer items to cater for greater working memory spans in selected samples. This can be easily achieved because the explanatory item response framework that forms the basis of the JaJ allows for automatic generation of additional longer items and estimation of their difficulty without recalibrating the item bank. A second limitation concerns the lack of validation of the JaJ with samples of children younger than 14 years of age; from the entire spectrum of academic achievement and those from clinical samples. The Automated Working Memory Assessment battery in which Mister X task was initially introduced [36] was designed for use with children as young as 5. Whilst it would be reasonable to assume that the JaJ can also produce meaningful results with children this young, it still needs to be confirmed empirically. A third limitation of the current study is a lack of power to establish norms (i.e., mean and the standard deviation for different ages and ability levels) for practical use in education as well as the generality of these correlations with other cognitive tasks. To do this, a validation with a larger and more heterogeneous sample would be necessary (see for example, Schrank, Mcgrew & Woodcock. [107]).

Finally, to address concurrent and divergent validity more completely, it would be necessary to formulate a larger study that measures different aspects of WM, visual spatial abilities, and intelligence simultaneously. Yet, this is beyond the scope of the present article. In general, whilst the JaJ task adequately reflects the theoretical definition of what a WM measure should consist of, more evidence about its sensitivity to other WM components is required (see for example, Miyake et al., who used nine tasks to investigate sensitivity of different tests [30]). Further research should include other WM tasks to investigate this.

In sum, we believe that the a-JaJ is a valid, reliable, yet flexible and easy-to-use tool for assessing the VSWM capacity of adolescents and adults in research contexts, with potential practical applications in education. It is freely available from https://github.com/klausfrieler/JAJ as an open-source package for the R software language and runs within the psychTestR package [51].

## Open practices statement

The anonymised datasets generated and analysed during the current study are available from the corresponding author on reasonable request. None of the studies was preregistered.

## Supporting information

S1 Appendix (DOCX)

S1 File (RAR)
