# Supplementary material for: The Jack and Jill Adaptive Working Memory Task: Construction, Calibration and Validation
Source: PLoS One. 2022 Jan 27;17(1):e0262200. doi: 10.1371/journal.pone.0262200 (PMC8794187; doi:10.1371/journal.pone.0262200)
Supplement: S1 Appendix — (DOCX) [file pone.0262200.s001.docx]

# **Appendix**

| **Table A1.** *IRT difficulty and discrimination parameters extracted from the explanatory IRT model described in Exp. 1.* | | | | |
| --- | --- | --- | --- | --- |
| Item ID | Length | L1 | Difficulty | Discrimination |
| A | 1 | 1 | -0.74 | 2.31 |
| B | 2 | 0 | 0.13 | 2.31 |
| C | 3 | 0 | 0.38 | 2.31 |
| D | 4 | 0 | 0.64 | 2.31 |
| E | 5 | 0 | 0.89 | 2.31 |
| F | 6 | 0 | 1.14 | 2.31 |
| G | 7 | 0 | 1.39 | 2.31 |
| Note: Parameters correspond to a 1PL model (e.g., [36]) where difficulty varies but the discrimination parameter is constant across items. Length denotes the length of the ball sequence and L1 is the dummy variable indicating sequences of length = 1. | | | | |
